# Potential Benefits to Dairy Cow Welfare of Using a Ceftiofur–Ketoprofen Combination Drug for the Treatment of Inflammatory Disease Associated with Pyrexia: A Field Clinical Trial on Acute Puerperal Metritis

**DOI:** 10.3390/ani11061597

**Published:** 2021-05-28

**Authors:** Isabella Lora, Mattia Massignani, Annalisa Stefani, Flaviana Gottardo

**Affiliations:** 1 Department of Animal Medicine, Production and Health, University of Padova, Viale dell’Università 16, 35020 Legnaro (Padova), Italy; isabella.lora@studenti.unipd.it (I.L.); mattiama92@libero.it (M.M.); 2 Istituto Zooprofilattico Sperimentale delle Venezie, Viale dell’Università 10, 35020 Legnaro (Padova), Italy; astefani@izsvenezie.it

**Keywords:** dairy cow, metritis, ceftiofur, NSAID, welfare

## Abstract

**Simple Summary:**

Some diseases of dairy cows require the use of an antimicrobial and an anti-inflammatory drug in association to be fully cured and relieve pain. However, pharmacological treatments in cattle are subject to strict regulations and restrictions, and cow handling is not always easy and safe. For these reasons, only the antimicrobial is often administered, thus not fully applying the appropriate therapeutic protocol. This study investigated whether the use of a drug combining ceftiofur and ketoprofen in a single injection instead of ceftiofur alone can improve the healing and welfare of dairy cows affected by a pyretic inflammatory disease, such as acute puerperal metritis. The results show that the variation in the physiological parameters was similar between the two treatment groups, and daily activity and milk yield did not differ from healthy cows. However, affected cows that were treated with the combined drug seemed to be more likely to become pregnant within 120 days in milk than those that received the antimicrobial alone, having an estimated number of days open more similar to that of the healthy cows.

**Abstract:**

This study aimed at investigating the benefits of using a drug combining ceftiofur and ketoprofen in a single injection on dairy cow welfare in the case of inflammatory disease with pyrexia, such as acute puerperal metritis (APM). Cows of an Italian dairy farm were examined between 5 and 14 days of calving: those with APM were randomly treated either with combined ceftiofur–ketoprofen (CD) or ceftiofur alone (C), starting from Day 0, and an equal number of healthy cows served as a control (CTR). Clinical examination and blood sampling were performed until Day 7 in each group according to specific schedules. Daily cow activity was recorded until Day 14 and daily milk production until Day 30. Additional data on fertility were collected until 120 days in milk (DIM). Data of 20 cows per group were analyzed. Body temperature and haptoglobin concentration dropped between Day 0 and 4 in both CD and C, approaching the level of CTR. The cure rate at Day 7 (body temperature < 39.0 °C) was 65 (CD) and 55% (C), without statistical difference. Neither cow activity nor milk production differed among the three groups. Reproductive performances in both CD and C were similar to CTR, but CD cows were 2.8 times more likely to be pregnant within 120 DIM than C, becoming pregnant about 14 days sooner. Both treatments (CD and C) have been effective in bringing the cows back to health conditions (CTR), and further studies would be needed to confirm the positive effect observed for CD on days open of the affected cows.

## 1. Introduction

Inflammatory conditions in dairy cows are responsible for cow depression, less locomotion, and reduced performances. Acute puerperal metritis (APM) is one of the most common and most serious inflammatory diseases during the puerperal period for the dairy cow [1]. It occurs typically within the first 4–10 days after parturition with a reported incidence rate ranging from 3 to 36% [1,2,3]. The condition is characterized by systemic signs of illness (fever ≥ 39.5 °C, anorexia, dehydration, depression, and reduced milk production) associated with a foul-smelling, brown-red, watery uterine discharge, with or without retention of fetal membranes [3,4,5]. The APM is due to bacterial contamination from the vagina and the environment into the uterus at parturition, where *Escherichia coli*, *Trueperella pyogenes*, *Fusobacterium* spp., and/or *Bacteroides* spp. are the most commonly isolated bacteria [1,5,6]. Bacterial infection of the endometrium induces an inflammatory response that causes delays in uterine involution and disrupts the survival of the embryo, in addition to pain responses [2,7,8]. As a consequence, APM can cause high economic losses, mainly related to low fertility, decreased milk production, milk withdrawal, increased health expenses, and premature culling of up to +30% compared with healthy cows [6,9,10].

Treatment of inflammatory conditions such as APM should therefore aim at reducing clinical signs (body temperature and signs of toxemia), inflammation, and pain, generally improving the wellbeing of the animal. The treatment of APM usually relies on local or systemic use of antimicrobials such as oxytetracycline, ampicillin, penicillin, and third-generation cephalosporins [1,2]. Ceftiofur, a third-generation cephalosporin, was offered early as a treatment for postpartum metritis in dairy cows thanks to its withdrawal period of zero hours for milk. Up to date, it is largely demonstrated that ceftiofur administered at 1 mg/kg per day by intramuscular or subcutaneous injection for 3 to 5 consecutive days is an effective antimicrobial option for the treatment of cows diagnosed with APM [4,6,11]. Due to the concerns on antimicrobial resistance in human medicine, ceftiofur is now considered a critically important antimicrobial by the World Health Organization [12] and should be used in veterinary medicine only for serious and motivated reasons [13]. However, third-generation cephalosporins are also considered critically important antimicrobials for veterinary medicine [14].

Being an inflammatory condition, anti-inflammatory drugs should be used in addition to antibiotics to reduce the symptoms associated with APM. The treatment of APM with non-steroidal anti-inflammatory drugs (NSAIDs) has been discussed in the literature, and it has been proven that some of them, such as ketoprofen, provide benefits in the treatment of APM and allow reducing the amount of antibiotic administered by half [15]. In the dairy practice, however, given the recognized efficacy of the antimicrobial treatment, the anti-inflammatory therapy risks being often skipped, because it means an additional drug to be bought and recorded in the official records, a withdrawal period (not for ketoprofen on milk [16]), and an additional injection to be performed.

For this reason, the on-market availability of a drug combining an antimicrobial (ceftiofur) and an NSAID (ketoprofen) in a single injection with zero withdrawal period for milk [17] seems of high interest for the dairy sector, as it has several advantages from practical, economical, bureaucratical (official recording of the treatment), and animal welfare points of view. The potential benefits of using this kind of drug for treating inflammatory diseases associated with pyrexia in dairy cows have still to be investigated.

For this purpose, APM was chosen as the target disease in the present study to test whether the use of the combined drug (ceftiofur + ketoprofen) instead of a commonly used therapy (ceftiofur alone) for the APM treatment can provide some additional benefits from the animal welfare point of view. The APM was chosen as the target disease because it is one of the most frequent and relevant inflammatory diseases associated with pyrexia in dairy cattle, it has been demonstrated that the inflammation associated with APM is painful [8], and the efficacy of both ceftiofur and ketoprofen for its treatment has already been proven [4,6,15]. The hypothesis was that the combined drug has positive effects on cow welfare by anticipating the recovery of the affected cows and thus allowing their rapid return to a healthy status and normal behavior, which is given by the healthy mates as a within-farm baseline reference. This has been evaluated by comparing physiological, behavioral, and productive parameters of the treated cows with a group of healthy cows in the same stage of lactation. Additionally, some reproductive parameters have been analyzed as further direct indicators of functional recovery and indirect indicators of animal welfare.

## 2. Materials and Methods

### 2.1. Study Sample

The study was carried out from November 2017 to March 2019 in a dairy farm located in northeastern Italy and rearing 370 Holstein cows. The farm was selected as it met the requirements for carrying out the field trial (number of cows, clinical history, equipment for herd monitoring) and based on the willingness of the farmer to be part of the study. The farm had loose housing with cubicles, a milking parlor with daily measurement of individual milk yield, and pedometers to measure cow activity. The feeding system consisted of total mixed ration distributed twice a day.

All the cows that calved during the study period (*n* = 526) underwent a clinical examination by a veterinarian between 5 and 14 days after parturition, excepting for May and June 2018 when the study was suspended due to the low number of expected calvings. The animals with a body temperature ≥ 39.3 °C were submitted to transrectal palpation of the uterus and those presenting reddish-brown fetid vaginal discharge or vaginal mucus containing white or off-white purulent material, were considered as having APM [6,18,19]. They were included and kept in the study if they had no vaginal lesions and history of cesarean or displaced abomasum operations, retained placenta, acute mastitis, lameness, or other diseases that required treatments before the clinical examination for APM detection or within 30 days after the inclusion in the study. These last conditions were necessary even to select the healthy cows to enroll as control, in addition to having no alteration at the clinical examination, normal post-partum uterine involution, no signs of APM, and a body temperature < 39.0 °C.

### 2.2. Study Design and Data Collection

The day of inclusion in the study was considered as Day 0. Cows diagnosed with APM were assigned to be treated either with a combination of ceftiofur and ketoprofen (Curacef^®^ Duo, Virbac, Carros, France) (CD) or ceftiofur alone (Ceftionil^®^, Iven, Madrid, Spain) (C) at 1 mL/50 kg of body weight (equal to 1 mg/kg of ceftiofur) for 3 consecutive days starting from Day 0 according to a randomization schedule produced by Excel^®^ using a simple randomization procedure. Curacef^®^ Duo is a marketed injectable combination of ceftiofur hydrochloride (50 mg/mL) and ketoprofen (150 mg/mL) registered in Italy for the treatment of bovine respiratory disease caused by *Mannheimia haemolytica* and *Pasteurella multocida* sensitive to ceftiofur, and for the reduction in clinical signs associated with inflammation or pyrexia in cattle. The treatment period was based on the drugs’ specific directions for use. The selected healthy cows were not treated and served as a control (CTR) for the study.

**Figure 1:**
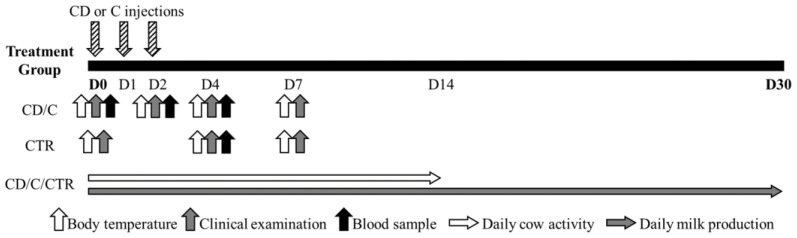
Cow monitoring protocol during the first 30 days (D) of the study period (CD = ceftiofur + ketoprofen; C = ceftiofur; CTR = control).

Clinical examination was performed on Days 0, 2, 4, and 7 in CD and C cows and on Days 0, 4, and 7 in CTR cows. Body temperature was measured at the same time of the day on Days 0, 2, 4, and 7 for cows in groups CD and C, and on Days 0, 4, and 7 for CTR cows (Figure 1). Cows were considered recovered when body temperature was < 39.0 °C, and depending on whether this condition was not reached within Day 7, an escape therapy could be adopted by the farm veterinarian (e.g., a different systemic antibiotic, etc.).

In order to investigate the effect of the treatment groups on the physiological response of the animals, haptoglobin was selected among the acute phase proteins as the main indicator of the inflammatory response in the case of APM [20,21,22], and cortisol was chosen as a widely recognized indicator of stress in animals [23]. For the assessment of serum haptoglobin and cortisol levels, blood samples were collected from the tail using a 10 mL Vacutainer^®^ tube (Becton Dickinson, Franklin Lakes, NJ, USA) on Days 0, 2, and 4 in CD and C cows. The CTR cows were sampled on Day 4 to determine baseline blood haptoglobin and cortisol levels (Figure 1). Blood samples were delivered to the laboratory (Istituto Zooprofilattico Sperimentale delle Venezie, Legnaro, PD, Italy) within 2 h after collection and were centrifuged at 1700× *g* for 10 min to collect serum. Serum haptoglobin was measured using the commercially available PHASE™ Haptoglobin Assay kit (Tridelta Development Limited, Maynooth, Ireland) applied to automated chemistry analyzers Cobas c501 (Roche Diagnostics, GmBh, Mannheim, Germany) according to the manufacturer’s instructions for automated method. The linearity range of the test was 0.5 to 250 mg/dL, the analytical sensitivity was 0.5 mg/dL, and intra and inter assay coefficients of variation (CV) were 5.3 and 5.7%, respectively. Serum cortisol was measured with a dedicated Cortisol II kit with the electrochemiluminescence immunoassay analyzer Cobas e601 (Roche Diagnostics): the linearity range was 1.5 to 1750 nmol/L, the analytical sensitivity was 1.5 nmol/L, and intra- and inter-assay CV were 3.9 and 9.0%, respectively.

Daily cow activity (number of steps per day) recorded by pedometers (Afiact Plus, Afimilk, Afikim, Israel) was collected from Day 0 to Day 14, and daily milk production (kilos of milk produced per day) recorded by the milk meters installed in the milking parlor (AfiMilk MPC, Afimilk, Afikim, Israel) was collected from Day 0 to Day 30 in all treatment groups (CD, C, and CTR; Figure 1). The farmer agreed to put the pedometers on the cows within two days of calving, which was earlier than he usually did.

Additionally, as direct indicators of functional recovery and indirect indicators of animal welfare, fertility data were collected during the first 120 days in milk (DIM) in the three treatment groups (CD, C, and CTR). They included the proportion of cows pregnant, pregnant at the first service, never served, and culled, as well as the time at first heat, first service, and pregnancy (i.e., last service before positive pregnancy diagnosis), and the number of services per cow and per pregnancy. The voluntary waiting period of the farm was 50 DIM; after that, all the cows in estrus were served (artificial insemination). The pregnancy diagnosis was performed by the farm veterinarian, who was blinded to the treatment group.

All the animal manipulations required in this study were performed by a veterinarian, who was blinded to the treatment group for the whole study period.

### 2.3. Sample Description and Data Analysis

The minimum sample size to carry out this study was five cows per group. This was calculated by *t*-test applied for differentiating healthy cows from cows with APM based on the reference limits of physiological parameters (serum haptoglobin concentration and body temperature) reported in the literature [21,24], and putting the type one error at 5% and the power of test at 90%. This method was chosen given the absence, to our knowledge, of published methods for calculating the minimum sample size required for differentiating between two treatment groups of animals affected by disease and for analyzing behavioral, productive, and fertility data of animals belonging to different treatment groups. However, for cautiousness, we put the minimum sample size required for this study at three times higher than the calculated, so at 15 cows per group.

Of the total 84 cows diagnosed as having APM, 67 were initially enrolled along with 35 healthy cows, according to the inclusion criteria of the study. However, due to either the occurrence of further diseases (e.g., mastitis, displaced abomasum, etc.), or culling, or technical incidents in sensor recordings within Day 30, 20 was the final number of cows analyzed per group (C, CD, CTR) and per parameter measured (physiological, behavioral, productive, and reproductive). The median parity of the cows was 2 for all the groups considered in the study, with a minimum of 1 and a maximum of 3 (C), 4 (CD), and 5 (CTR).

Statistical analyses were performed using SAS 9.4 software (SAS Institute Inc., Cary, NC, USA) and the level of significance was set at *p* < 0.05. In the case of daily data on cow activity and milk production, outliers (±3 standard deviations from the mean) were removed from the dataset in order to exclude potential errors in the automatic recording system. Physiological parameters, activity, milk production, and fertility data were compared between the three treatment groups (CD, C, and CTR) as well as between the groups CD and C only.

In order to compare the physiological parameters of CD and C groups with the baseline represented by the CTR group, the body temperature of CTR cows on Day 2 was calculated as the mean of the body temperature on Days 0 and 4, whereas the values of serum haptoglobin and cortisol measured on Day 4 were reported also for Days 0 and 2. Because haptoglobin and cortisol were not normally distributed, they were log-transformed to perform the analysis. Due to the high number of missing values, Day 0 was excluded from the analysis of cow activity and milk production.

A general linear model for repeated measurements was used to compare the variations in the physiological parameters (body temperature, haptoglobin, and cortisol), cow activity, and daily milk yield between treatment groups. Parity (primiparous vs. multiparous cows) and the number of DIM on Day 0 were included in the model as covariates, and the Bonferroni correction was applied. Cow activity and daily milk yield (var) were included in the model as “score of variation”, that is, after transformation to a uniform scale (varying from 0 to 1) made by the equation proposed by Steensels et al. [25]:

$$\text{score of variation}=\frac{\text{var}-\text{minimum (var)}}{\text{maximum (var)}-\text{minimum (var)}}$$

The percentage of cows recovered (i.e., with body temperature < 39.0 °C) on Days 2, 4, and 7 in groups CD and C were compared by *z*-test. Percentages of cows pregnant, pregnant at first service, never served, and culled within 120 DIM were compared among groups by a Chi-square test with the Marascuilo approach. The number of services per cow and the number of services per pregnancy within 120 DIM were compared among treatment groups by the non-parametric Kruskal–Wallis test with the Dwass–Steel–Critchlow–Fligner method.

The comparison of the number of DIM at pregnancy (i.e., DIM at last service before positive pregnancy diagnosis) between groups was performed by survival analysis with the Bonferroni correction (Kaplan–Meier survival plot and Cox proportional hazard model).

## 3. Results

The incidence rate of APM observed in this study was 16.0% of the total number of cows examined (*n* = 526).

### 3.1. Physiological Parameters

**Figure 2:**
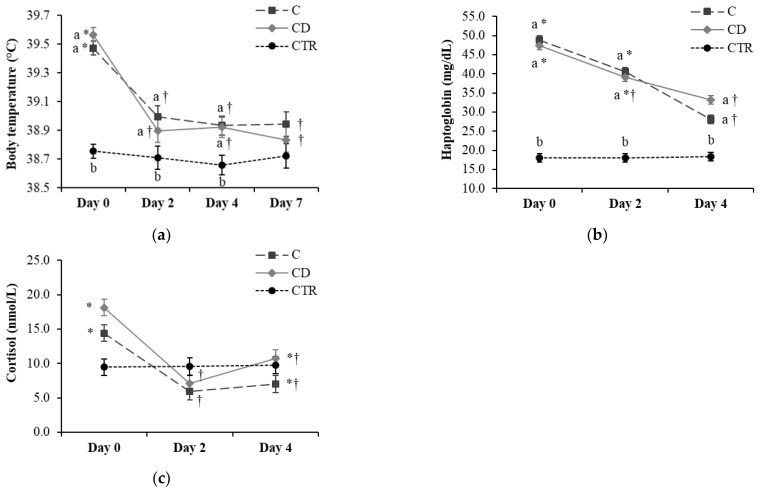
Evolution over time of (**a**) body temperature, (**b**) haptoglobin, and (**c**) cortisol serum concentration in the three treatment groups of 20 cows each (C = ceftiofur; CD = ceftiofur + ketoprofen; CTR = control). Least square means and standard errors as resulted from the general linear model are reported. ^a,b^ Different letters indicate differences between the three groups (C, CD, and CTR) over time (*p* < 0.05). *,† Different symbols indicate differences within the groups C and CD over time (*p* < 0.05).

The average body temperature did not differ between groups CD and C on Day 0 but was higher than the body temperature in CTR cows (*p* < 0.001; Figure 2a). Average body temperature evolution over time was similar in groups CD and C, with a drop on Day 2 compared with Day 0 (*p* < 0.001), and a flat trend in the following days. However, average body temperatures in CD and C cows were higher (*p* < 0.05) than in CTR cows until Day 7, when body temperature became similar in all groups, going below 39.0 °C.

On Day 0, serum haptoglobin concentrations were similar between groups CD and C and higher than the CTR group (*p* < 0.001; Figure 2b). Serum haptoglobin levels steadily decreased over time in both groups CD and C, and were lower on Day 4 vs. Day 2 in group C (*p* < 0.001) and lower on Day 4 compared to Day 0 in both CD and C groups (*p* < 0.001). Despite this drop, haptoglobin concentrations in groups CD and C were still higher than in the CTR group on Day 4 (*p* = 0.002).

The serum cortisol levels did not differ among the three groups over time (Figure 2c). In both groups CD and C, serum cortisol concentrations were lower on Day 2 compared with Day 0 (*p* < 0.05), with an intermediate value on Day 4.

The percentage of cows cured on Days 2, 4, and 7 did not differ between groups CD and C (*p* > 0.10). In terms of absolute values, however, the percentages of cows cured in the CD group were higher than those in the C group on Days 2 and 7 (60 vs. 40% and 65 vs. 55%, respectively), whereas there was no difference on Day 4 (60% each; results not shown). Based on the choice of the farmer and the farm veterinarian, none of the cows included in the treatment groups (CD and C) underwent any escape therapy.

### 3.2. Activity and Milk Production

**Figure 3:**
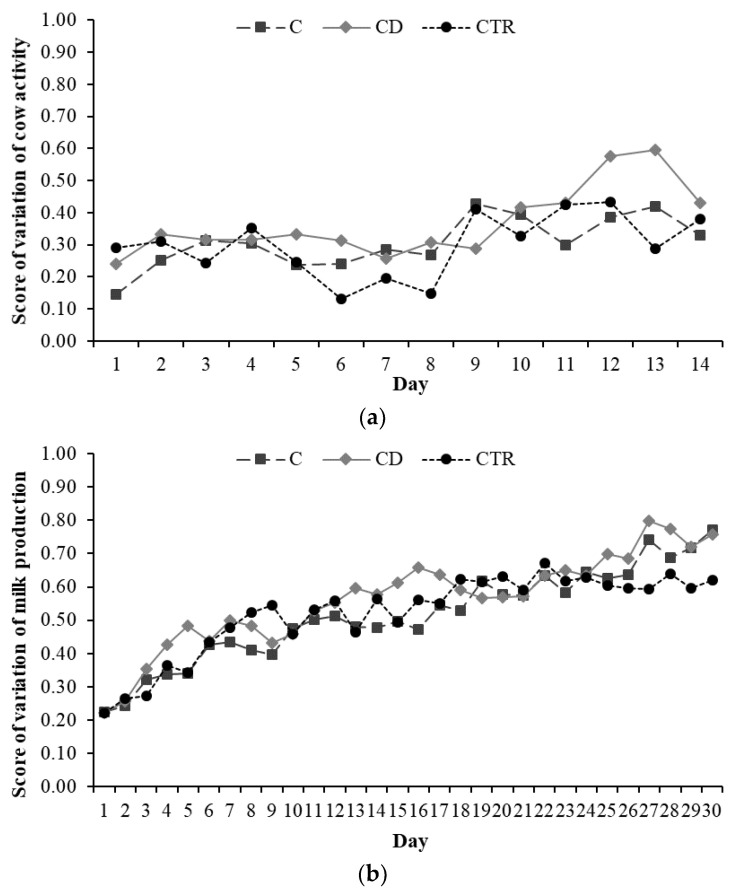
Evolution over time of cow (**a**) daily activity and (**b**) milk production in the three treatment groups, C (ceftiofur), CD (ceftiofur + ketoprofen), and CTR (control), of 20 cows each, expressed as score of variation (range 0 to 1; *p* > 0.10).

Treatment affected the score of variation of neither cow activity nor daily milk yield (*p* > 0.10; Figure 3).

### 3.3. Fertility Data

**Figure 4:**
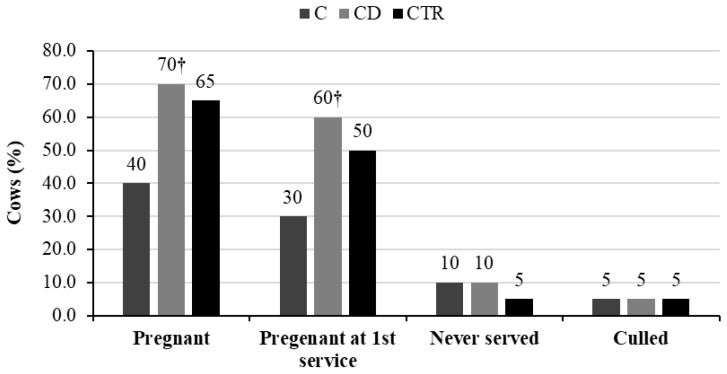
Percentages of cows pregnant, pregnant at first service, never served, and culled within 120 days in milk over the total number of cows (*n* = 20) included in each of the three treatment groups (C = ceftiofur; CD = ceftiofur + ketoprofen; CTR = control). † Values of groups C and CD differed at *p* < 0.10.

No significant differences were found in the proportion of cows pregnant, pregnant at first service, never served, and culled between the three groups within the observation period of 120 DIM. However, the percentages of cows pregnant and pregnant at first service were almost double in group CD compared with group C (*p* = 0.057; Figure 4).

Even if CD cows seemed to show better performances regarding the number of services per cow and per pregnancy within 120 DIM than C ones, no statistically significant differences were detected amongst groups (Table 1).

**Figure 5:**
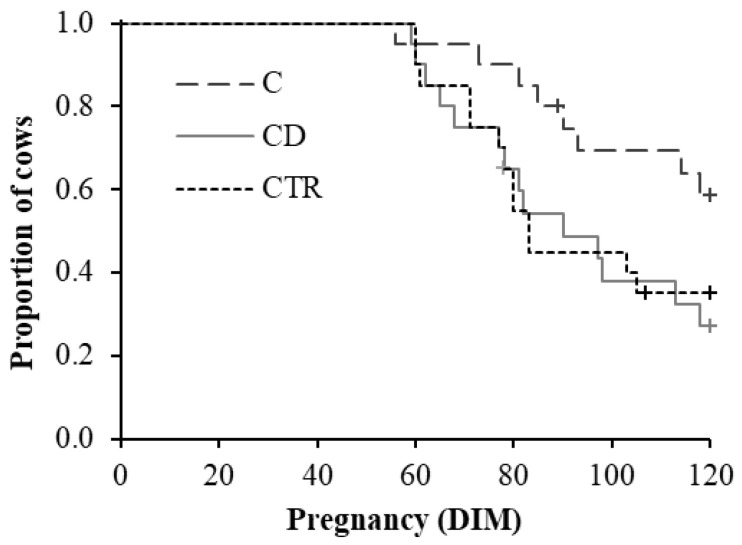
Kaplan–Meier survival plot showing the proportion of cows over DIM (days in milk) that became pregnant within 120 DIM in the three treatment groups (C = ceftiofur; CD = ceftiofur + ketoprofen; CTR = control) of 20 cows each. Censored values are reported (+).

Treatment did not affect DIM at pregnancy when comparing all the groups (CD, C, and CTR; Wilcoxon test of equality over strata: χ^2^ = 5.30, *p* = 0.071), but a difference arose when only C and CD groups were compared (Wilcoxon test of equality over strata: χ^2^ = 4.48, *p* = 0.034; Figure 5).

Particularly, cows of CD and CTR groups became pregnant 14 and 19 days sooner, respectively, than cows of the C group (Table 2). Moreover, CD cows were 2.8 times more likely to be pregnant within 120 DIM than C ones (Table 2).

## 4. Discussion

This study aimed at evaluating the benefits on cow recovery and welfare of using a drug that combined an antimicrobial and an anti-inflammatory substance in a single injection, instead of an antimicrobial alone, in cases of inflammatory disease associated with pyrexia, for which the use of the combined drug was authorized in Italy. Acute puerperal metritis in dairy cows was chosen to run this study because it is an inflammatory disease with pyrexia that is of interest for the dairy sector, which can be objectively diagnosed and classified, and for which the effectiveness of ceftiofur treatment has been already demonstrated [4,6,11].

A recent systematic review regarding diagnostic methods for APM demonstrated that elevated rectal temperature (fever) and fetid, watery, reddish-brown vaginal discharge were described in 39 and 21 of 48 peer-reviewed research papers, respectively, addressing APM [26]. Particularly, the body temperature threshold used in previous controlled studies evaluating the efficacy of ceftiofur in cows with APM varied from 39.2 [18] to 39.5 °C [3,6]. Therefore, the inclusion criteria adopted in this study were consistent with the best available reference definition of APM.

The body temperature significantly improved in the two treatment groups (CD and C) since Day 2 and returned to values comparable to that in the CTR group on Day 7. Even if it was not possible to detect any difference in body temperature trend over time between CD and C groups, the percentage of cows cured (T° < 39.0 °C) on Day 2 was higher in CD cows (+20%) than in C. Again, this difference was not significant, but the clinical cure rate in the group CD after a 3-day treatment, and as soon as Day 2, was similar to those (65%) observed in other studies on Day 7 or 14 after treatment with ceftiofur for 5 consecutive days [4,27]. These observations seem to point out a higher rapidity of action when ceftiofur is associated with ketoprofen. However, a higher number of cows per group would have been needed to confirm or deny this result. Furthermore, other studies showed the risk of overestimating the clinical cure rate when the rectal temperature was used alone as a determination of cure [6,24]. Thus, redefining the clinical cure rate in this study by adding other clinical signs (e.g., vaginal discharge) may have led to different results.

Acute-phase proteins are molecules recognized as indicators for acute inflammatory processes in animals and, among them, haptoglobin is reported as the main indicator of inflammation associated with APM in cattle [21,22]. In the present study, values in groups CD and C on Day 0 were considerably higher than the cut-off of 18.5 mg/dL defined by Khoshvaghti et al. [21] for APM detection, even if they were lower than the average value showed by cows with APM in the same study (89.0 mg/dL). Serum haptoglobin levels in the group CTR were in line with the value of 10.8 mg/dL found in other studies for clinically healthy cattle [21] but did not reflect the physiological postpartum increase (up to 80 mg/dL) described in other reports [28]. The significant differences in haptoglobin concentrations in cows with and without APM in this study on Day 0 confirm that this acute-phase protein is a valuable indicator for acute inflammatory processes. At last, the significant decrease in serum haptoglobin levels in our study of about 18 mg/dL from Day 0 to Day 4 in groups CD and C, confirmed the effectiveness of ceftiofur in reducing acute inflammation consistently with the results of other studies [18,21,29]. Nevertheless, the absence of statistical differences observed between the two treatment groups did not allow us to detect, unexpectedly, a further beneficial effect that could be attributed to the association with ketoprofen.

Cortisol is the primary hormone routinely used as an indicator of the level of stress and pain an animal is experiencing, including that linked to health perturbations [30,31]. In this study, however, no conclusion regarding the evolution of stress could be drawn because serum cortisol concentrations did not significantly differ between cows with APM and healthy cows on Day 0, and no statistical significance was detected between the two treatment groups during the observation period. However, it is interesting to note that the evolution of serum cortisol levels of cows with APM showed a similar trend as that of body temperature, with a significant decrease on Day 2 and then a flat evolution or a slight increase until Day 4.

Surprisingly, cow activity and daily milk yield were not impacted by the disease at the study start and no statistical differences were observed between the two treatment groups regarding these two parameters. Indeed, results from other studies showed that activity was decreased in cows diagnosed with clinical metritis [32]. Similarly, numerous reports associated APM with a lower milk yield [3,5,18]. It has also to be noticed, however, that the effect of the disease on cow milk production and activity is mainly observed in the few days before disease diagnosis [25,33], but it is difficult to detect after that, probably due to the start of the pharmacological treatment. In this study, no effects were noticed in the few days after APM diagnosis, and data available from calving to APM diagnosis were not enough to give reliable results. It is also true that alterations before the APM diagnosis and treatment were not a specific aim of this study.

The absence of statistical differences in fertility data, such as the proportion of cows pregnant, pregnant at first service, never served, and culled, as well as the average number of services per cow and per pregnancy within 120 DIM between the treatment groups (C and CD) and the CTR group of this study, indicated that both treatments were effective in restoring the reproductive function of cows. Such effectiveness has undoubtedly been helped by the early APM detection allowed by the controlled study and was in line with previous reports [34]. Drillich et al. [6] showed a positive correlation between metritis and culling that was not possible to detect in this study. Differently from Drillich et al. [6], however, in this study cows culled before Day 30 were excluded from the dataset, and cows not served within 120 DIM were not considered as culled; therefore, the lack of effect on the proportion of cows culled was somehow expected.

On the other hand, Giuliodori et al. [35] reported that non-treated cows with puerperal metritis had a lower risk for pregnancy within 100 DIM compared with healthy cows, and affected cows treated with ceftiofur were more likely to become pregnant by 100 DIM than non-treated cows. These results were in line with the findings of the current study, where treated cows did not differ from healthy ones in terms of average DIM at pregnancy. However, when reproductive performances of only CD and C groups were compared, the results showed that cows in the group CD were significantly more likely to become pregnant within 120 DIM than C ones. This interesting result could be attributed to a beneficial effect of the association with ketoprofen, and can positively affect the economic return of the herd. Indeed, Inchaisri et al. [36] estimated a net economic loss ranging from EUR 0.76 to 1.95 per cow per day extra for days open and calving interval. In the case of CD cows, days open were reduced by about 14 days compared with C ones, which means an economic saving of about EUR 10.64 to 27.30 per cow.

It is also worth noticing that, due to the limited number of cows included in the study groups, the results on the reproductive performances of the cows found in this study should be considered with wariness.

## 5. Conclusions

This study investigated the benefits of administering a combined drug containing an antimicrobial and an anti-inflammatory instead of an antimicrobial alone in cases of inflammatory disease with pyrexia in dairy cows. Acute puerperal metritis was chosen as the target disease and several physiological, behavioral, productive, and reproductive parameters of cows were recorded. The use of the combined drug did not lead to different trends in physiological parameters (serum haptoglobin and cortisol concentration, and body temperature), cow activity, and milk production compared with the use of the antimicrobial alone, both allowing cow recovery and having a similar trend with respect to the healthy cows that served as a control. However, it was observed that cows with APM that were treated with the ceftiofur–ketoprofen combination were considerably more likely to become pregnant within 120 DIM than cows with APM that were treated with ceftiofur alone, with similar performances to the healthy cows. Given the effectiveness of the combined drug in the treatment of an inflammatory disease associated with pyrexia, further studies would be needed to confirm the positive effect observed on the reproductive performances of the affected cows.

## Figures and Tables

**Table 1:** Descriptive statistics of the number of services per cow and per pregnancy within 120 days in milk in the three treatment groups of 20 cows each (CD = ceftiofur + ketoprofen; C = ceftiofur; CRT = control). Results from the Kruskal–Wallis test are reported too.

	Mean ± SD	Median	Min	Max	*p*-Value
Services per cow (n)					
CD	1.15 ± 0.67	1.0	0	3	0.240
C	1.50 ± 0.83	1.5	0	3	
CTR	1.30 ± 0.66	1.0	0	3	
Services per pregnancy (n)					
CD	1.29 ± 0.61	1.0	1	3	0.959
C	1.38 ± 0.74	1.0	1	3	
CTR	1.31 ± 0.63	1.0	1	3	

**Table 2:** Least square means (LSM) and standard errors (SE) of days in milk (DIM) at pregnancy in the three treatment groups, CD (ceftiofur + ketoprofen), C (ceftiofur), and CTR (control), of 20 cows each, as resulted from the survival analysis. Hazard ratio (HR) and 95% CI of becoming pregnant within 120 DIM, as resulted from the Cox proportional hazard model for both the comparisons (CD and C vs. CTR, and CD vs. C), are reported too.

		CD and C vs. CTR			CD vs. C		
Pregnancy (DIM) ^1^	LSM ± SE	HR	95% CI	*p*	HR	95% CI	*p*
CD	92.2 ± 5.2	1.10	0.52–2.34	ns	2.75	1.14–6.67	*
C	106.1 ± 4.5	0.41	0.17–0.99	†	1.00	–	
CTR	87.4 ± 4.0	1.00	–				

^1^ DIM at last service before positive pregnancy diagnosis. † = *p* < 0.10; * = *p* < 0.05; ns = *p* > 0.10.

## Data Availability

The data presented in this study are available on request from the corresponding author.
